# Evaluation of the “Burgenland PREvention trial of colorectal cancer Disease with ImmunologiCal Testing” (B-PREDICT)—a population-based colorectal cancer screening program

**DOI:** 10.1186/s12876-024-03242-7

**Published:** 2024-04-30

**Authors:** Stefanie BREZINA, Gernot LEEB, Andreas BAIERL, Evelyn GRÄF, Monika HACKL, Philipp HOFER, Harald LANG, Michaela KLEIN, Karl MACH, Remy SCHWARZER, Wilhelm WLASSITS, Andreas PÜSPÖK, Andrea GSUR

**Affiliations:** 1 Center for Cancer Research, Medical University of Vienna, Borschkegasse 8a, Vienna, 1090 Austria; 2 Department of Internal Medicine, Hospital Oberpullendorf, Oberpullendorf, Austria; 3 Department of Statistics and Operations Research, University of Vienna, Vienna, Austria; 4 Institute of Clinical Pathology and Microbiology, Oberwart, Austria; 5 Statistics Austria, Vienna, Austria; 6 Department of Pathology, Medical University of Vienna, Vienna, Austria; 7 Outpatient Clinic for Medical and Chemical Laboratory Diagnostics, Eisenstadt, Austria; 8 Austrian Cancer Aid Burgenland, Bad Sauerbrunn, Austria; 9 Department of Internal Medicine II, St. John’s Hospital, Eisenstadt, Austria

**Keywords:** Colorectal cancer, Two-stage screening, FIT

## Abstract

**Background:**

The colorectal cancer (CRC) screening program B-PREDICT is a population based invited two stage screening project using a faecal immunochemical test (FIT) for initial screening followed by a colonoscopy for those with a positive FIT. B-PREDICT was compared with the opportunistic screening colonoscopy (OPP-COL), performed in course of the nationwide screening program.

**Methods:**

Within B-PREDICT all residents of the Austrian federal state Burgenland, aged between 40 and 80 are annually invited to FIT testing. All individuals who underwent initial colonoscopy in Burgenland between 01/2003 and 12/2014, were included in this study. Individuals from the FIT-triggered invited screening program B-PREDICT were compared with those from the non-FIT triggered OPP-COL.

**Results:**

15 133 individuals from B-PREDICT were compared to 10 045 individuals with OPP-COL. CRC detection rates were 1.34% (CI-95%, [1.15; 1.52]) in B-PREDICT compared to 0.54% in OPP-COL (95%-CI, [0.39; 0.68] *p* < 0.001). The decrease in the age standardized incidence rates of CRC was more pronounced in the population screened with FIT than in the general population screened with colonoscopy. Changes in incidence rates per year were -4.4% (95%-CI, [-5.1; -3.7]) vs. -1.8% (95%-CI, [-1.9; -1.6] *p* < 0.001).

**Conclusions:**

B-PREDICT shows a two-fold higher detection rate of CRC as well as HRA compared to OPP-COL.

## Background

Colorectal cancer (CRC) is the second leading cancer related cause of death worldwide and represents a major public health issue [1]. In Austria, the CRC incidence rate is observed in the lower third within the European Union with about 49.2 per 100.000 inhabitants each year [2]. CRC is a complex disease with both genetic and environmental factors contributing to individual risk of CRC. The natural history of sporadic CRC usually involves slow progression of about 10 to 15 years from precancerous polyps to cancer, which offers opportunities for screening and early detection [3]. Early detection of CRC is an important issue since stage at diagnosis remains the most important prognostic factor [4]. As CRC is one of the most preventable cancers, population-wide screening programs have the potential to detect early precancerous lesions, thereby contributing to the reduction of CRC mortality and morbidity by earlier diagnosis and treatment [5–7].

Therefore, European guidelines for CRC screening (ESMO clinical practice guideline for diagnosis, adjuvant treatment and follow-up, 2010) recommend that persons aged between 50 – 74 years should be screened by guaiac-fecal occult blood test (gFOBT) or fecal immunochemical test (FIT) annually. Nowadays, FIT-test is the preferred approach in testing for occult blood in feces used for colorectal cancer screening programs [8]. If tested positive a clinical follow-up using colonoscopy should follow [9].

In Austria, screening for CRC with screening colonoscopy was included in the remuneration scheme of mandatory health insurance in 2005. Inhabitants aged above 50 years have the opportunity to undergo colonoscopy every ten years and to perform a FIT test annually. However, only a small proportion (*15.4 – 16.8%)* of the relevant targeted group aged between 50 – 75 years take the opportunity of this opportunistic program (OPP-COL) [10].

Currently, no nation-wide organized invited screening program for CRC exists in Austria. However, two federal states of Austria, Vorarlberg and Burgenland, have established invited CRC screening programs. In Vorarlberg, a colonoscopy-based screening program was initiated in 2007 inviting all insured inhabitants aged above 50 years to undergo a complete colonoscopy.

In 2002 the federal state Burgenland had the highest age-standardized incidence rates of CRC in Austria [2], mainly attributed to unhealthy lifestyle factors. Therefore, an invited population-based two-step screening program "The Burgenland PREvention trial of colorectal cancer Disease with ImmunologiCal Testing" (B-PREDICT) was initiated in 2003. Annually, inhabitants of Burgenland aged between 40 and 80 are invited to participate in this program using FIT as an initial screening. Participants with a positive FIT are offered further diagnostic work-up with a complete colonoscopy.

To evaluate the efficacy of B-PREDICT, the invited FIT triggered screening program, was compared with the nationwide opportunistic colonoscopy program (OPP-COL).

## Patients and methods

### B-PREDICT

"Burgenland PREvention Trial of colorectal cancer DIsease with ImmunologiCal Testing" (B-PREDICT). B-PREDICT is a two-stage screening project where more than 150 000 inhabitants of Burgenland aged between 40 and 80 are invited annually to participate in this program using a Fecal Immunochemical Test (FIT) as an initial screening. Positive tested individuals (≥ 10 µg haemoglobin / g faeces) are offered a complete colonoscopy. All endoscopists performing colonoscopies are participating in the nationwide quality assurance program for colonoscopy [11]. B-PREDICT was initiated from Karl Mach in a part of Burgenland (Oberpullendorf) 2003, since 2006, B-PREDICT is conducted in the whole province Burgenland.

The IT-center of the social health insurance company of Burgenland identifies the target group and coordinates the project. FITs (OC-Sensor®, Mast Diagnostica, Germany) are send per mail to all participants and they are asked to return the test at a local doctor’s office. All stool samples are analyzed by a central laboratory (Outpatient Clinic for Medical and Chemical Laboratory Diagnostics, Eisenstadt, Austria). General practitioners (registered doctors) are responsible for documentation of FIT results and refer patients with positive FIT test to colonoscopy. All physicians, who perform colonoscopy, are participating in the nationwide quality assurance program for colonoscopy.

The cut-off level for a positive FIT result was a hemoglobin concentration ≥ 50 ng hemoglobin /mL corresponding to ≥ 10 μg hemoglobin/g feces. The qualitative stool testing was used from 01/2003 to 12/2009. Since January 2010 a quantitative system, without changing the threshold for a positive result, replaced the qualitative test.

The provincial government of Burgenland grants the financial support.

### Opportunistic screening colonoscopy

Austria has included an opportunistic colorectal cancer screening program in the remuneration scheme of mandatory health insurance in 2005. Individuals aged above 50 years are offered a screening colonoscopy every ten years and faecal occult blood test starting by the age of 40.

In the province of Burgenland both screening modalities, B-PREDICT as well as the not FIT triggered opportunistic screening colonoscopy (OPP-COL) are provided to patients.

### Study design

All individuals, who underwent initial colonoscopy in Burgenland between 01/2003 and 12/2014 were included in the study. Individuals younger than 40 years, those with clinical symptoms or those with history of inflammatory bowel disease or a hereditary predisposition were excluded. The study population comprised of two groups, namely the invited FIT triggered screening program (B-PREDICT) and the not FIT triggered opportunistic screening colonoscopy.

### Endoscopic findings

Patients were classified into four groups according to the pathological and endoscopic results: CRC, high-risk adenomas (HRA), low-risk adenomas (LRA) and “other endoscopic findings”. The group of CRC also included patients with microinvasive carcinomas. HRA were defined as tubular adenomas > 1 cm, sessile serrated adenomas, adenomas with villous patterns or high grade intraepithelial neoplasia. In contrast, tubular adenomas ≤ 1 cm were considered as LRA. Individuals with other colonoscopic findings include all patients diagnosed with hyperplastic polyps and diverticula as well as those without any pathologic findings.

### Colorectal cancer incidence

CRC incidence and mortality data were obtained from the Austrian National Cancer Registry and the Austrian Causes of Death Statistics. The Austrian National Cancer Registry is a population-based registry operated by Statistics Austria, Causes of Death Statistics is also generated by Statistics Austria. Both statistics are produced on a legal basis, based on mandatory notifications.

Data in both statistics are coded using ICD-10, data extracted for this study included all cases coded C18-C21. The timestamp for cancer registry data was 2019/12/09.

The CRC incidence rates from 1983 to the year 2017 of Burgenland were compared to the rest of Austria. The region of Vorarlberg was excluded because it has a colonoscopy-based CRC screening model since 2007.

### Statistics

Measures of central tendency and dispersion were calculated for demographic data and characteristics of colonoscopies. Group differences of patients with B-PREDICT and OPP-COL were carried out using two-sample t-tests for continuous variables and χ2-tests for dichotomous variables.

Frequencies of endoscopic findings were compared between B-PREDICT and OPP-COL groups by applying separate χ2-tests for each endoscopic finding against all patients without this endoscopic finding. Confidence intervals for proportions were derived for all incidences of endoscopic findings.

Developments of incidence rates over time were analyzed by linear regression. Separate models were fitted for the time periods before and after the start of B-PREDICT (1983 -2002 and 2003 -2017). Differences in slopes were tested using interaction effects between region and time (mean centered).

All tests were two-sided and *p* values less than 0.05 were considered statistically significant. Distributional assumptions for t-tests were checked visually by quantile–quantile. All statistical analyses were performed with the statistical software R version 4.3.1 [12].

### Ethics approval and consent

The present study was performed in accordance with the Declaration of Helsinki in its current edition. The study protocol was approved by the ethical review board “Ethikkommission Burgenland’’ (approval number: EK 97/2019) and written informed consent was obtained from all patients included in the study.

## Results

### Patient characteristics

Between 2003 and 2014*,* 204,516 individuals received 1,504,340 FITs. 115,677 individual subjects returned 539,348 FITs. This reflects a rate of 56.6% patients that have participated in B-PREDITC at least once. 43,586 (8.1%) of 539,348 returned FITs were tested positive. These 43,586 positive FITs were returned by 31,267 individual subjects, corresponding to 27.0% of individuals who at least received once a positive FIT result. A colonoscopy within one year after a positive FIT result was documented in 22,231 cases, corresponding to 18,355 individuals of these 31,267 individuals (58.7%). For the further analysis the first documented colonoscopy per individual within the observation period was considered. This reduced the number of patients within the B-PREDICT group to 15,567. After applying exclusion criteria, a final number of 15,133 B-PREDICT patients were included and compared to 10,045 OP-COL individuals**.**

Patient characteristics of the B-PREDICT group, as well as of the OPP-COL group are presented in Table 1. Individuals of the B-PREDICT group were significantly older and presented significantly more often with diverticula. Within the B-PREDICT group, the mean time span between positive FIT result and colonoscopy was 72 days. Furthermore, the majority of colonoscopies was performed in hospitals compared to doctor’s offices.

**Table 1:** Characteristics of individuals with FIT-triggered colonoscopy (B-PREDICT) compared to individuals with screening colonoscopy (OPP-COL)

	**B-PREDICT *N* = 15 133**	**OPP-COL *N* = 10 045**	***p*-value**
Age in years;	61.5	60.6	< *0.0001*
(Mean, SD)	(10.8)	(10.2)	
Gender Males	8 028	5 282	*0.4680*
(n; %)	53%	52%	
Mean days between FIT and colonoscopy (SD)	72.3 (57.1)	n.a	
Time between positive FIT and colonoscopy (n; %)			
- < 1 month	3 152 (21%)	n.a.	
- 1–2 months	4 468 (29%)	n.a.	
- 2–3 months	3 454 (22%)	n.a.	
- 3–4 months	2 054 (14%)	n.a.	
- 4–5 months	841 (6%)	n.a.	
- 5–6 months	414 (3%)	n.a.	
- 6–12 months	750 (5%)	n.a.	
Colonoscopy performed in doctor’s office (n; %)	3 546 (23.4%)	1 707 (17.0%)	< *0.0001*
Diverticula (n; %)	4 350 29%	2 736 27%	*0.0096*

*SD* Standard deviation, *FIT* Fecal immunochemical test, *N.a* Not applicable

### Detection rates of adenomas and CRC

The detection rates within the B-PREDICT and OPP-COL group are presented in Table 2.

**Table 2:** Distribution of endoscopic findings by screening model

	**B-PREDICT *N* = 15,133**	**OPP-COL *N* = 10,045**	***p*-value**
CRC (95%-CI)	*N* = 202 1.33% (1.15%; 1.52%)	*N* = 54 0.54% (0.39%; 0.68%)	< *0.0001*
HRA (95%-CI)	*N* = 2 143 14.17% (CI: 13.62; 14.73)	*N* = 686 6.83% (6.34%; 7.33%)	< *0.0001*
LRA (95%-CI)	*N* = 2 565 16.95% (CI: 16.36; 17.56)	*N* = 1 296 12.90% (12.26%; 13.57%)	< *0.0001*
Hyperplastic polyps and normal findings (95%-CI)	*N* = 10 223 67.55% (66.78%; 68.28%)	*N* = 8 009 79.73% (78.93%; 80.5%)	< *0.0001*

*CRC* Colorectal cancer, *CI* Confidence interval, *HRA* High-risk adenoma, *LRA* Low-risk adenoma, *B-PREDICT* Organized FIT-triggered colonoscopy, *OPP-COL* Opportunistic screening colonoscopy, *N* Number of cases

The detection for CRC and HRA was in the B-PREDICT group two-fold higher than in OPP-COL. However, for patients older than 70 years, CRC detection rates was similar.

**Fig. 1:**
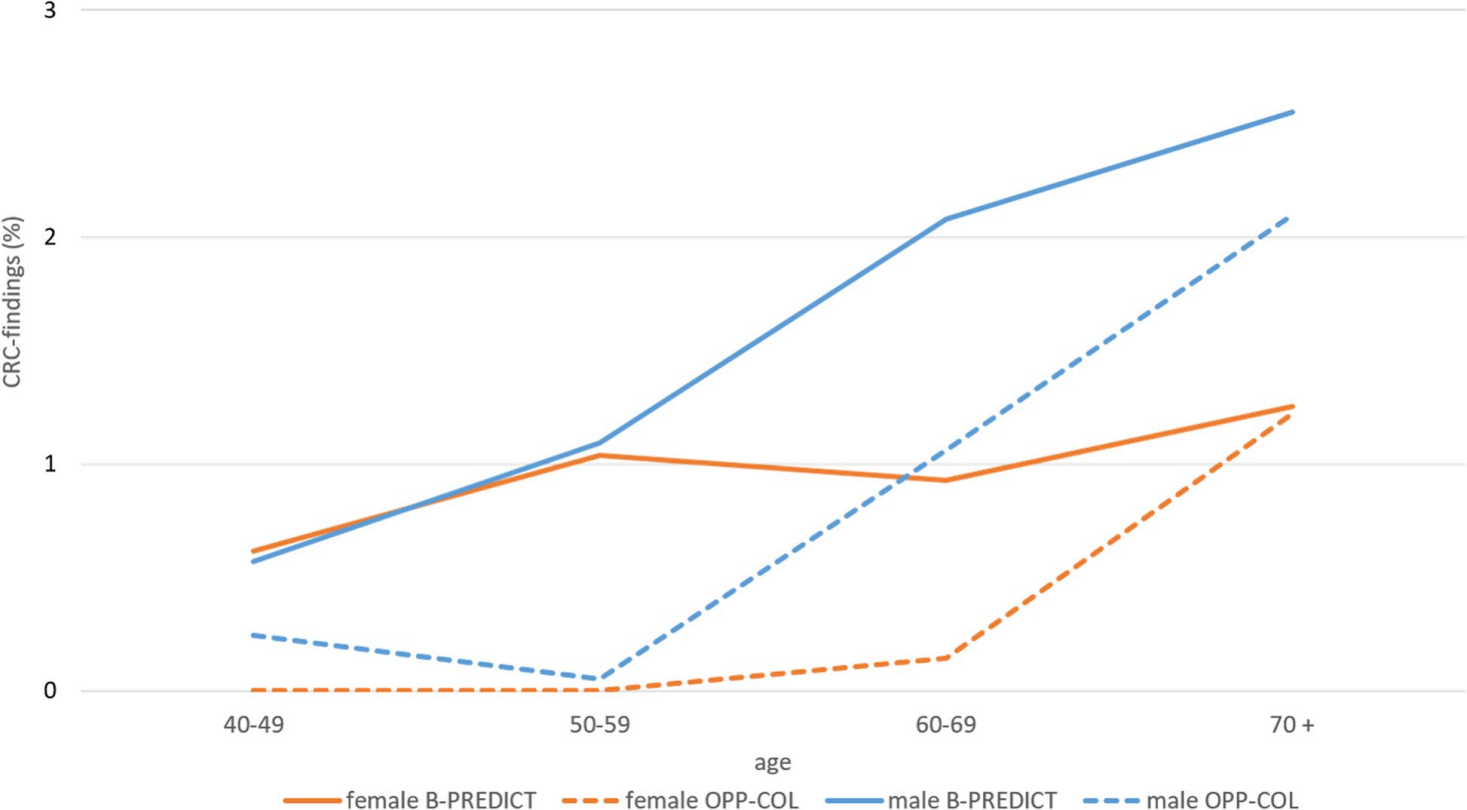
Proportion of colorectal cancer (CRC) findings by screening model, age and gender. Orange lines show CRC findings for female patients, blue lines indicate findings for males. Dotted lines illustrate findings in the OPP-COL population. Consistent lines show data for the B-PREDICT patients

**Fig. 2:**
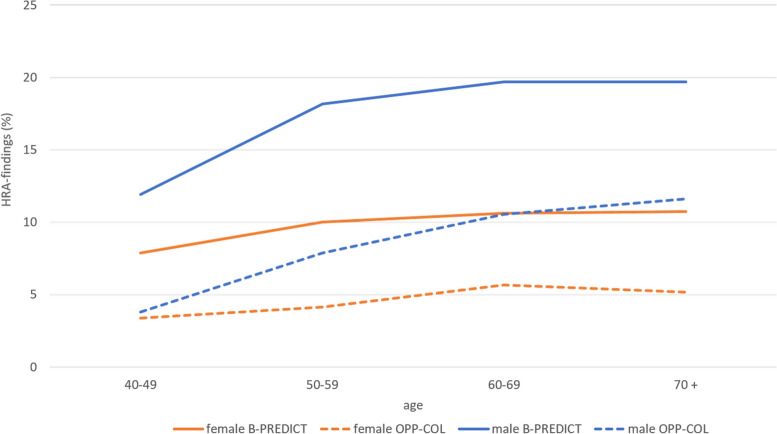
Proportion of high-risk adenoma (HRA) findings by screening model, age and gender. Orange lines show CRC findings for female patients, blue lines indicate findings for males. Dotted lines illustrate findings in the OPP-COL population. Consistent lines show data for the B-PREDICT patients

In general, detection rates of CRC, HRA and LRA increase with age and are higher in males than in females. Detection rates of HRA and LRA are higher within the B-PREDICT group compared to the OPP-COL group, regardless of age and gender (detection rate of LRA not shown). This can also be seen in patients with CRC, aged between 40 and 70. However, this difference becomes less obvious in patients older than 70 years (Figs. 1 and 2).

### Impact of FIT on CRC-incidence

Between 1983 and 2002, the CRC incidence rate of the province of Burgenland was higher compared to the other Austrian federal states with 85.5 per 100 000, versus 76.7 per 100 000. During this period, rates in Burgenland as well as in the rest of Austria neither increased nor decreased significantly (*P* = 0.456 and *P* = 0.260).

In 2003, the population size of the province of Burgenland was 135,170 inhabitants aged between 40 and 80 years (being included in the B-PREDICT screening) and 79,709 inhabitants aged between 50 and 75. For the rest of Austria there were 3,544,847 inhabitants as well as 2,092,361 inhabitants aged 40 to 80 years as wells as 50 to 75 years, respectively. In 2014, this figures raised in Burgenland to 154,792 and 98,810 inhabitants aged 40 to 80 years as wells as 50 to 75 years, respectively. For the rest of Austria inhabitants numbers increased to 4,087,517 inhabitants aged between 40 and 80 and 2,561,264 individuals between 50 and 75 years.

**Fig. 3:**
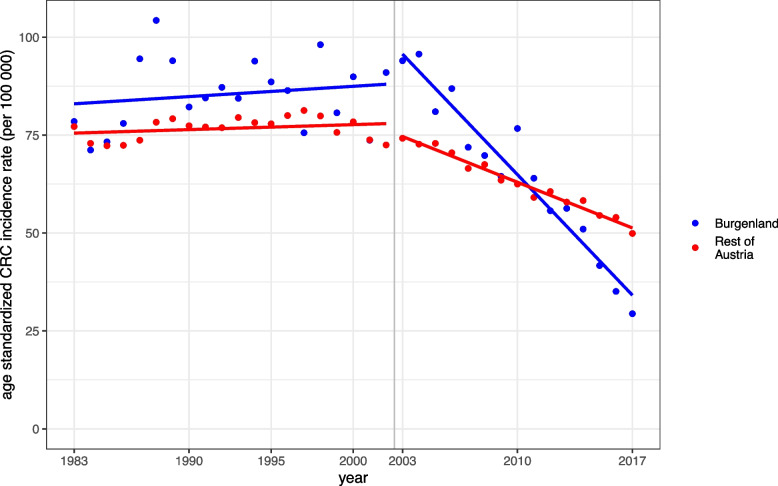
Age standardized incidence rate of colorectal cancer in Burgenland (blue line) and the rest of Austria (red line). FIT screening is applied in the province of Burgenland, while opportunistic screening colonoscopy is recommended in the rest of Austria (without the region of Vorarlberg)

Between 2003 and 2017, CRC incidence rates decreased in Austria (-1.8 units per year, 95%-CI [-1.9. -1.6]) with a much more pronounced decline for Burgenland (-4.4 units per year, 95%-CI, [-5.1; -3.7], P for difference in decrease between Burgenland and rest of Austria: < 0.001). Since 2012, these trends lead to even lower rates for Burgenland than for the rest of Austria. In 2017 the age-standardized incidence rate for Austria was 49.2 while it was just 29.4 for Burgenland (Fig. 3) [2].

## Discussion

A large proportion of CRCs are highly preventable. Modifiable risk factors are Western-type dietary (i.e. high intakes of fats, red/processed meats, refined grains, sugary foods, alcoholic beverages, and low intakes of dietary fibre, fruits, vegetables) and unhealthy lifestyle habits (overweight, obesity, physical inactivity [13, 14]. Beside these opportunities for primary prevention, secondary prevention is an important issue. In countries with long-standing screening programmes such as the Netherlands or England, CRC incidence has decreased substantially [15, 16]. In the Netherlands, biannual fecal immunochemical test screening for individuals aged between 55–75 started in 2014 [17]. After five years of implementation of this nationwide screening program a decrease in stage II and IV CRC incidence was observed. Furthermore, stage III and IV patients had less extensive disease and improved survival rates.

Colonoscopy is the gold standard to reduce CRC incidence and mortality as it allows detection and removal of precursor lesions and early-stage tumors directly during the examination [18]. However, the participation rate is rather low because of the invasive screening procedure and bowel preparation. Although colonoscopy represents a widely used method of screening for CRC, diagnosing colorectal disease, and treating colorectal mucosal lesions it is a complex process that offers several opportunities for misadventures and complications like bleeding or colon perforation. The incidence of post-colonoscopy complications increases in elderly patients or patients with inflammatory bowel diseases [19]. However large-scale studies using big data for post-colonoscopy complications have illustrated perforation rate of 0.005–0.085% and a post-colonoscopy bleeding occurrence of 0.001–0.687% [20].

B-PREDICT, a two-stage invited screening program, is using the noninvasive FIT test as initial test to preselect individuals for follow-up colonoscopy. Participants who test positive for presence of fecal occult blood are offered a diagnostic colonoscopy. FIT screening is relatively cheap, easy to use and noninvasive but suboptimal sensitive, leading to false positive and negative results. FIT is currently the best available noninvasive CRC screening tool.

Starting age of screening programs is a major point of discussion. As increasing CRC incidence was observed in individuals younger than 50 in the last years a potential adjustment of screening guidelines must be considered in some countries [21, 22]. B-PREDICT has included participants from a relatively young age of 40. A recent review by Saraiva et al*.* reporting on early onset CRC could show that screening in Austria contributed to the decreased in early onset CRC incidence in Austria. They report that Austria is one of three European countries (together with Italy and Lithuania) where early onset CRC decreased in the last 10 years. Furthermore, they showed that two (Austria and Italy) of the only three European countries where early onset CRC has declined have screening programs that begin below 50 years old [23].

CRC incidence was decreasing markedly in Burgenland within the last decade. This decrease may mainly attribute to the invited screening program B-PREDICT as we could show a two-fold higher detection rate of CRC and HRA compared to the opportunistic screening colonoscopy, performed in course of the nationwide screening program. We could show that the B-PREDICT screening outperform the national screening program in terms of CRC as well as HRA detection rate and in terms of effectiveness by decreasing the incidence of CRC. Furthermore, the annual invitation using a two-stage strategy allowing patients to perform the initial test at their private homes makes B-PREDICT an easy-access offering to patients of every age group.

High participation rates of CRC screening programs are of great importance to reduce the incidence and mortality of CRC [24]. B-PREDICT achieved an acceptable participation rate of 56.6%, comparable to other invited screening programs such as National Screenings in the Netherlands, Lampang (Thailand) or Northern Italy (49.7%—68%) [25–27].

The latency for diagnostic colonoscopy is an important marker for a well-functioning screening, beside adenoma detection rate and participation rate. The majority of patients included in the present study were able to complete the diagnostic colonoscopy within 3 months after positive FIT. Performing colonoscopy more than 90 days after positive FIT was shown to correlate with the number of CRC diagnosed, advanced stage disease and presence of multiple HRA [28].

However, a significantly increased risk for CRC was only seen more than 9 months after a positive FIT in a large US cohort [29]. Consequently, the logistics around CRC screening are an issue, which should not be underestimated. A national program in the Netherlands recently faced an important bottleneck of CRC screening [27]. After study initiation, it became evident that the anticipated data (e.g. attendance to the program, positivity rates of FIT, detection rates of advanced adenomas/ CRC) assessed during the planning phase differed considerably from real track data, which threatened the feasibility of the screening program. There were too many false positive FIT results with the initially defined cut-off value, which surpassed the logistic capacity. In order to address this problem, a microstimulation screening analysis was initiated predicting a similar number of CRC deaths prevented even when a higher threshold for a positive FIT result was applied [27]. This highlighted that the choice of cut-off value for positive FIT should be made according to the availability of follow-up colonoscopy resources [27, 30, 31]. Our study demonstrated that the implementation of FIT is able to increase the detection rates of HRA and CRC, and at the same time to reduce CRC-incidence in the long-run. Due to the high acceptance of FIT in the targeted age group more individuals may be reached without affecting the colonoscopy capacity, which remains a good of limited accessibility.

Moreover, an Austrian study recommends that men may earlier undergo screening colonoscopy for CRC, as male gender was shown to be significantly associated with higher prevalence of adenomas [32]. Our findings support this recommendation as we observed higher detection rates of CRC, HRA and LRA in males compared to females. However, detection rates of HRA and LRA were higher within the B-PREDICT group compared to the OPP-COL group, regardless of gender.

The main limitation of the present study is that patients were not randomized to either the B-PREDICT screening population or the opportunistic screening group making the study population heterogeneous However, due to the large comprehensive dataset established in course of the B-PREDITC screening a defined assignment of patients to either the B-PREDICT screening or OPP-COL group is possible and allows for a valid analysis and comparison of both screening strategies within one population. Furthermore, the present study is limited to one province of Austria, namely the province of Burgenland being the only federal state with an invited CRC screening program. However, due to the great success of the B-PREDICT program and the results of the presented data, there are currently several considerations to initiate a nationwide invited FIT-triggered CRC screening. Furthermore, the diagnostic value of negative FIT results were not assessed so far. However, due to the comprehensive database created throughout the implementation of B-PREDICT further analysis and validation will be conducted.

## Conclusion

In the present study we could show that age standardized incidence rates of CRC was more pronounced in the B-PREDICT population pre-screened with FIT than in the population included through opportunistic CRC screening. The CRC detection rate was doubled in patients of the B-PREDICT screening program compared to the national wide opportunistic CRC screening.

Therefore, we conclude that the implementation of an organized two-staged screening program using a FIT pre-screening appears to reduce the age standardized CRC incidence rates more efficiently than an opportunistic colonoscopy screening.

## Data Availability

The datasets generated and/or analyzed during the current study are not publicly available as they present clinical as well as medical data embedded in a clinical information system but are available from the corresponding author on reasonable request.

## Abbreviations

CRC: Colorectal cancer
B-PREDICT: Burgenland PREvention trial of colorectal cancer Disease with ImmunologiCal Testing
FIT: Fecal immunochemical testing
OPP-COL: Opportunistic screening colonoscopy
HRA: High-risk adenomas
LRA: Low-risk adenomas
